# Ethical aspects of diagnosis and interventions for children with fetal alcohol Spectrum disorder (FASD) and their families

**DOI:** 10.1186/s12910-017-0242-5

**Published:** 2018-01-05

**Authors:** Gert Helgesson, Göran Bertilsson, Helena Domeij, Gunilla Fahlström, Emelie Heintz, Anders Hjern, Christina Nehlin Gordh, Viviann Nordin, Jenny Rangmar, Ann-Margret Rydell, Viveka Sundelin Wahlsten, Monica Hultcrantz

**Affiliations:** 1 Stockholm Centre for Healthcare Ethics (CHE), Department of Learning, Informatics, Management and Ethics, Karolinska Institutet, Stockholm, Sweden; 2 Swedish Agency for Health Technology Assessment and Assessment of Social Services (SBU), Stockholm, Sweden; 3 Medical Management Centre, Department of Learning, Informatics, Management and Ethics, Karolinska Institutet, Stockholm, Sweden; 4 Clinical Epidemiology, Department of Medicine, Karolinska Institutet and Center for Health Equity Studies, Stockholm, Sweden; 5 Department of Neuroscience, Psychiatry, Uppsala University, Uppsala, Sweden; 6 Center of Neurodevelopmental Disorders, Karolinska Institutet (KIND), Stockholm, Sweden; 7 Department of Psychology, University of Gothenburg, Gothenburg, Sweden; 8 Department of Psychology, Uppsala University, Uppsala, Sweden; 9 Department of Neuroscience, Child and Adolescent Psychiatry, Uppsala University, Uppsala, Sweden; 10 Department of Learning, Informatics, Management and Ethics, Karolinska Institutet, Stockholm, Sweden

**Keywords:** Alcohol consumption, Alcohol exposition, ARBD, ARND, Diagnostics, Ethics, Family support, Fas, FASD, pFAS

## Abstract

**Background:**

Fetal alcohol spectrum disorders (FASD) is an umbrella term covering several conditions for which alcohol consumption during pregnancy is taken to play a causal role. The benefit of individuals being identified with a condition within FASD remains controversial. The objective of the present study was to identify ethical aspects and consequences of diagnostics, interventions, and family support in relation to FASD.

**Methods:**

Ethical aspects relating to diagnostics, interventions, and family support regarding FASD were compiled and discussed, drawing on a series of discussions with experts in the field, published literature, and medical ethicists.

**Results:**

Several advantages and disadvantages in regards of obtaining a diagnosis or description of the condition were identified. For instance, it provides an explanation and potential preparedness for not yet encountered difficulties, which may play an essential role in acquiring much needed help and support from health care, school, and the social services. There are no interventions specifically evaluated for FASD conditions, but training programs and family support for conditions with symptoms overlapping with FASD, e.g. ADHD, autism, and intellectual disability, are likely to be relevant. Stigmatization, blame, and guilt are potential downsides. There might also be unfortunate prioritization if individuals with equal needs are treated differently depending on whether or not they meet the criteria for a specific condition.

**Conclusions:**

The value for the concerned individuals of obtaining a FASD-related description of their condition – for instance, in terms of wellbeing – is not established. Nor is it established that allocating resources based on whether individuals fulfil FASD-related criteria is justified, compared to allocations directed to the most prominent specific needs.

## Background

Alcohol consumption during pregnancy can affect the development of the brain of the fetus during differentiation and growth. *Fetal alcohol spectrum disorders* (FASD) is an umbrella term covering several defined conditions, including Fetal Alcohol Syndrome (FAS), partial FAS (pFAS), Alcohol-Related Birth Defects (ARBD) and Alcohol-Related Neurodevelopmental Disorder (ARND) [1]. These conditions have in common that they are etiologically based, i.e., alcohol consumption during pregnancy is taken to play a causal role in the development of these conditions.

The FAS diagnosis spans over a complex set of physical, behavioral, and cognitive deviations. Partial FAS (pFAS) shares the complexity and deviations of FAS, but not all criteria for a FAS diagnosis are (fully) present. The ARBD condition describes individuals with minor or major physical deviations, but without behavioral or cognitive impairments, whereas ARND instead describes an atypical cognitive development and behavioral deviations among individuals with normal physical development. ND-PAE is a condition very similar to ARND, potentially identifying an almost identical set of individuals [1].

Individuals meeting the criteria for the different conditions under the FASD umbrella experience a number of cognitive, physical, behavioral, and social problems that affect their everyday lives in a negative way [2–4]. Parents to children with FASD feel burdened by the difficulties to handle their everyday family life, social isolation, and worry for their child’s future. They also experience a lack of knowledge, understanding, and support among professionals in health care and in the social services [4–7].

However, the value for individuals of being identified as having a condition within FASD remains controversial. In this paper, ethical aspects for and against using the perspective of alcohol etiology are explored by researchers who are independent of the controversies in this field. The aim was to answer three overarching ethical questions:

1. Is diagnosing FASD conditions ethically problematic? If so, in what ways?
2. Are interventions for children and their families based on such conditions ethically problematic?
3. What are the direct and indirect consequences on health and wellbeing of diagnosing children with FASD conditions?

## Methods

This paper is based on work performed within a health technology assessment on FASD conducted by the Swedish Agency for Health Technology Assessment and Assessment of Social Services (SBU) [8]. SBU is a Swedish authority with the commission to make independent assessments of medical and social interventions. The assessments include systematic reviews of effectiveness as well as analysis of ethical and health economic aspects. In 2015–2016 SBU, together with of a number of experts in the field (authors of this paper), conducted systematic reviews of the prevalence of disabilities for individuals with FASD and the effects of interventions for the children and their families [8]. During this work, ethical aspects relating to diagnostics, interventions, and family support were identified and discussed.

Studies relevant to this paper were identified in a semi-structured way. This was not a formal systematic review since there were no pre-specified inclusion criteria, and several steps were carried out by a single reviewer. The aim was to make sure that relevant overarching ethical aspects were not missed, not to identify all published studies in the area. The electronic literature search focused on the populations within FASD and was also used for the systematic reviews on prevalence and interventions mentioned above (for the detailed search strategy, see [8]). The literature search was performed by an information specialist and included the databases Cinahl, Cochrane, EMBASE, ERIC, PsycInfo, PubMed, SocIndex, Sociological Abstracts & Social Services Abstracts, and Scopus. The search covered studies published up to October 1, 2016. Abstracts published after January 1, 2000 (2043 abstracts) were screened by two reviewers independently and 430 abstracts were identified as potentially relevant for a discussion on ethical aspects. 78 of the identified abstracts were read in full by the first author and 39 of these were included as literature to be used in the ethical analysis, based on the recognition that they provided information that was sufficiently relevant to be included. A late complementary search added one paper.

The identification, systematization, and further discussion of ethical aspects relating to FASD were carried out in the tradition of analytical ethics [9] and applied ethics [10, 11]. Very broadly described, the various issues were analyzed in two different respects by responding to two basic types of questions: Is there anything good/right or bad/wrong in itself with this phenomenon? And what about the consequences – is the balance of positive and negative aspects on the whole good or bad? In responding to the broad questions relating to FASD addressed in this paper, the mapping and analysis of relevant issues has benefited considerably from repeated in-depth discussions within the expert group of the FASD project and with the help of relevant literature. Input to preliminary versions of the ethical analysis was provided by the SBU council (a scientific advisory board) and a reference group of medical ethicists (listed under Acknowledgments). The reflections on ethical and social aspects also benefitted from a synthesis of qualitative studies describing living with FASD conditions, from the perspectives of parents and patients, carried out by the FASD project group [8]. A check-list for systematic identification of ethical aspects of healthcare technologies was used as an additional support [12].

## Results

### Potential advantages of FASD-related diagnostics

For the individual with the condition and the rest of the family, a diagnosis or clear description of the condition can have valuable consequences of several kinds, positively influencing their wellbeing and potentially having preventive effects:

- A clear description of the condition gives the individual and the family an explanation of the problems and difficulties encountered [13].
- Communication within and outside the family is facilitated by having a label, a name for the condition. For instance, having a diagnosis can make it easier to get in touch with others experiencing similar difficulties, such as patient organizations and social media [8].
- Knowing that the child is not capable of handling the situation better than he or she in fact does, can make it easier to remain constructive in situations that occur [6, 14].
- It can be valuable for other family members to know that the child with FASD is not always in control of his/her behavior and that, for instance, fits of rage can occur without there being dislike or genuine anger towards other family members [6, 14].
- If a diagnosis or described condition is established in a healthcare practice, a consequence might be that children with disabilities are identified earlier, which in turn might lead to greater preparedness for the family as well as for kindergarten and school, health care, and social services; examinations may get initiated earlier, family support may be in place earlier, etc [14].
- Conditions under the FASD umbrella are associated with motor problems, sight and hearing problems, limited cognitive abilities, attention and concentration difficulties, limited impulse control, mood swings, autism, and social disabilities [8]. If the cognitive and social disabilities are accepted to be related to FASD, the diagnosis or description of the condition might give further examinations a clearer direction.
- It may also move attention from simply focusing on distinct difficulties, to actually considering the greater picture of the child’s abilities and difficulties. With such an overview, it might also be easier to find strategies to better cope with the challenges of everyday life.
- Information to parents that their child suffers from FAS may prevent them from having further children with the same condition. Parents to a child with suspected FASD who does not fulfill the criteria for FAS could be informed that the impairments may be caused by prenatal alcohol exposure. In these cases, fetal damage due to alcohol exposure cannot be proven, because today there are no biomarkers clearly establishing the causal relationship in the individual case. However, the parental awareness of alcohol being a possible factor may contribute to increased cautiousness with alcohol consumption during future pregnancies.

### The importance of a diagnosis/condition description in order to get help

Some children with FASD have specific patterns of delayed or deviant development so that criteria are fulfilled for one (or more) of the neurodevelopmental disorders, e.g. ADHD, autism, or intellectual disability. These diagnostic terms imply that there are certain underlying neuropsychological deficits, and might be of help when deciding on the most effective adaptation of the learning situation, and the best forms of parent information and training. However, many children and adults with FASD do not have all the symptoms needed to get any of these diagnoses; they may, nevertheless, have severe problems in daily life.

The literature implies that obtaining a diagnosis may be the key that opens doors to different resources in health care, school, and social services [4, 6, 15]. In other words, acquiring the diagnosis might have a series of positive consequences. Reports from some countries witness that without a diagnosis, there is no access at all to special resources, while the presence of a diagnosis provides access to resources, such as medication, expert consultations, special support in school, family support, and better contact with the social services [15, 16]. The variety of diagnoses that are essential to resources differs from society to society. Today, FAS is included in the medical diagnostic classification system, but not FASD; the term FASD just conveys that alcohol is the cause of, or one of the causing factors, of the disorder in an individual. Further diagnostic evaluation might be needed to convey specific resources.

However, even when a diagnosis has been given and there are obvious needs that require attention in order for the person and his/her family to function reasonably well in daily life, there is no guarantee that the help will be available. It seems to be a common view among parents with a child with FASD that understanding and support from professionals is still largely missing [4–6].

### Potential disadvantages of FASD-related diagnostics

There are also a number of potential disadvantages with FASD-related diagnoses. A distinction needs to be made between FAS and the wider FASD spectrum, since FAS is a medical diagnosis with fairly well-defined criteria, while the boundaries to normality and to other disorders are less clear for the other entities within FASD.

A first set of concerns regard using FASD as a diagnosis in itself: FASD as a diagnosis in itself would be of little use since “knowing that someone has FASD does not specify which symptoms they have”. Therefore the typical link between diagnosis and a reasonably well specified set of treatments is missing, which clearly diminishes the point of the diagnosis [17]. A general FASD diagnosis would still have the point of placing the individual in a context of related disabilities and difficulties. Apart from that, there would be no positive consequences for the concerned individuals, unless resources were allocated based on the diagnosis.

### Stigmatization, blame, and guilt

Because the FASD-related conditions are etiological (i.e., they are including its cause as part of the understanding of the condition), there are concerns about FASD-related diagnostics regarding negative consequences of stigmatization, blame, and feelings of guilt:

- It may cause feelings of guilt among biological parents, especially mothers [4, 17, 18].
- It may create problems within the family due to blame-putting between parents [5].
- It may create problems for the family in its relations to others due to blame-putting, for instance, from professionals [5] and from foster parents [17, 18].
- The concerned children, and their mothers/families, may be stigmatized. (There are many parallels in the literature to other areas where there is a ‘social etiology’ of the health issue at hand, such as being a parent with a drug addiction [19], or stigmatization in relation to congenital HIV [20]).

Whether blame, stigmatization, and feelings of guilt are likely effects of diagnostics will at least partly depend on the nature of information being spread about the condition, and what aspects of this information will be picked up by different recipients; for instance, whether or not it will become widely accepted that the fetus may be harmed by alcohol even before the mother is aware of her pregnancy status, in which case people might be less inclined to blame the mother.

### Unfortunate prioritization effects?

Another kind of criticism concerns the potential risk that focus on a FASD diagnosis or FASD conditions, instead of on specific needs, may lead to a reduction of attention paid to children with similar disabilities, but who do not meet the FASD criteria. In other words, there is a risk that children with equal needs will either be sorted in a group that is paid more attention than before, the FASD group, or in a group of “others” that may be paid even less attention than before. If none of these groups were given sufficient attention before, it would still be a step forward that one of them receives sufficient attention after a new FASD-related scheme is introduced. However, it remains highly problematic from a fairness perspective whether individuals with equal needs are treated unequally in health care. This is also against legal regulations and prioritization guidelines in, for instance, Sweden. Hence, it is possible that a better approach would be to focus on the greatest difficulties in both groups, regardless of diagnosis, for instance those related to cognitive disabilities, reduced impulse control, and difficulties with concentration and memory. With a maintained focus on a FASD condition, it is of course important which condition is chosen, since the four different conditions mentioned above are described in different ways and identify different sets of individuals with different disabilities and needs. Again, fairness and sound prioritization is at stake.

### Diagnostics and autonomy

There seem to be both advantages and disadvantages from the perspective of autonomy when concerned individuals are diagnosed. Autonomy presupposes decision-making capacity [21]. If autonomy is understood as the individual’s right to make decisions about things that are of particular concern to him- or her (the right to autonomy) and the degree of control that the individual has over his or her own life, then a diagnosis can promote autonomy by increasing self-understanding and, hence, to some degree, the ability to be in command of one’s own life. Early diagnoses can increase the chances of obtaining early support that strengthens the individuals’ abilities over time and in that way strengthen the control over their own lives.

But the diagnosis can also be a burden that reduces the concerned individuals’ confidence in the possibilities of shaping their own lives, for instance by leading to resignation regarding their own ability to reach personal goals. If the diagnosis leads to stigmatization beyond what is caused by how others perceive the disabilities, this can also restrict the individuals’ space to manoeuvre, because of the practical social effects of stigmatization. In that way it can harm autonomy. Then the question arises which effect dominates. An earlier SBU report on ADHD showed that most of the respondents perceived being given a diagnosis as positive [22]. However, it is unclear how relevant this is for FASD-related diagnoses, since they relate to presumed or confirmed alcohol consumption during pregnancy, which may lead to increased stigmatization of the mother.

For the sake of cautiousness, several national authorities recommend total abstinence during pregnancy [23–25]. Out of respect for the autonomy of fertile women, it is important that it is made clear what knowledge and other considerations this recommendation is based on.

### The value of FASD for choosing interventions for children and their families

Studies have shown that persons with FASD experience that their cognitive, mental health, and social difficulties affect their daily life in a limiting way [3, 4]. A number of studies have also shown that many parents experience ignorance, lack of information, and lack of support from, for instance, the social services [5, 17]. It seems to be a relatively common experience among parents that they have had to fight hard to receive any help at all [5, 13]. This might lead to suffering in the form of worry and the feeling of being abandoned by society.

However, although voices have been raised that it is important that children with FASD conditions obtain a diagnosis, diagnostics seem to have a limited impact on choice of interventions – it does not give much guidance beyond what the separate difficulties and disabilities do [9, 17, 18]. The developmental difficulties met by children with FAS, pFAS, and ARND overlap with symptoms found in children with ADHD, autism, and intellectual disabilities. Methods that are validated for training and habilitation of children with these neurodevelopmental disorders have been adapted for use in groups of children with FASD. The cooperation with parents has been described as vital in these studies. A few methods specifically applied to FASD were found in the review of interventions performed at SBU [8], e.g. increased support to foster families, and group sessions for teenagers and their parents to decrease the risk of alcohol misuse. Yet there is no form of habilitation care that is shown to have a *specifically positive* effect on children with a FASD condition. Hence, there is a limited advantage with providing a FASD-related diagnosis in this respect. However, as in every case of disability, it is important to have a good knowledge of the background neuropsychological problems in the individual child to choose the best intervention. Sometimes, evaluation concerning possible FASD may lead to better testing and more knowledge of positive factors and problems, and therefore, better habilitation care.

To conclude, there are no or few specific training methods described in the literature, but this is not to say that there is nothing to do for children with a FASD condition. To the contrary, many approaches can be taken to train them to improve different skills, such as training of memory and learning strategies, language and reading training, math training, training of social skills, and computer-based training of executive skills, among others. There is also medication for certain conditions, such as ADHD. Family support can potentially have multiple positive effects. It may allow for the family to feel recognized, and not abandoned with its problems, which can be perceived as a help as such. More hands-on support can include education, help with social training of the child, respite care, and financial support.

## Discussion

There are both potential advantages and disadvantages associated with FASD-related diagnostics. In all cases of developmental disorders, there are complex and co-acting background factors; these can consist of direct toxic effects from alcohol exposure, other environmental/social factors, as well as genetic factors [26]. We acknowledge the obvious role of alcohol as cause of the disorder for some of the individuals described as having FASD. Ethical problems arise in other cases, where the causal link is not well established, but rather assumed. For similar reasons, statistics about the number of cases belonging to the wider spectrum is an uncertain ground for claims about the need for prevention.

The concerns about the assumed causal link between alcohol exposure and functional disabilities relate to a higher degree to specific conditions under the FASD umbrella, especially the ARND condition: formally, it only claims that there is an association between previous consumption of alcohol and the concerned disabilities, yet it is built around the idea of a causal link [27]. If the causal interpretation becomes established practice among clinicians, which has been claimed to already be the case in some places, then a recognized risk factor is turned into the (perceived) causal factor [27]. A similar argument has been raised in relation to the broad and unspecific FASD label [17]. What this criticism suggests is that interpretations of diagnoses and conditions may arise in clinical practice that deviate from established definitions and that may influence how individual patients and their families are treated, for instance, in their encounters with health care. In practice, this may mean that the biological mothers, in a context where definitions are not strictly applied, might be accused of alcohol consumption during pregnancy and that this behavior has caused the disabilities of the child, also when none of this is true [17, 27, 28].

In addition, the focus on alcohol consumption in the definitions of the conditions might lead to an underestimation or lack of attention regarding other potential causal factors. Since not all women who have consumed alcohol during pregnancy give birth to children who fulfil criteria for a FASD condition, and some women who have not consumed alcohol during pregnancy have children with symptoms consistent with a FASD condition, it is obvious that other factors also play a role in the development of these conditions [17, 26, 27, 29]. It would be valuable if these other factors were also given proper attention, which could lead to better understanding of causes behind the functional and social disabilities.

Some critics argue that by merely focusing on the effects of fetal alcohol exposure, social problems related to harmful drinking, such as economic and social inequality and social marginalization, are turned into a problem for the individual. By focusing on individuals, the moral responsibility can be placed on ‘bad mothers’ instead of on society’s institutions and their failure to protect and support this vulnerable group [29, 30]. Even if children with FASD are receiving help under such a regime, the bigger picture and the long-term solutions relating to the need for changes in the structure of society may remain unidentified. This criticism, thus, concerns both unfair blaming of the individual and insufficient societal responsibility for its most vulnerable citizens.

In light of this discussion, it is worth noting that the pros and cons described, as well as their relative weights, are dependent on active choices in society. For example, whether or not receiving a FASD related diagnosis is required to get access to special resources and support will depend on choices made within e.g. the health care system or the individual school. The consequences of receiving a FASD related diagnosis also depend on contextual factors, such as the attitudes and perspectives within society – factors that through active choices can change over time.

### Limitations

We did not make a formal systematic review to identify relevant publications, which can be seen as a weakness, since it reduces transparency and the potential for the search to be reproduced. Studies were identified in a semi-structured way, with the aim to make sure that relevant overarching ethical aspects were not missed, but without the ambition to identify all published studies in the area and without pre-specified inclusion criteria. We estimate that if ethical analyses explicitly relating to diagnosis or interventions for FASD had been carried out during the time-period relevant to our literature search, then our search would have identified them. However, the search strategy does not guarantee that there are no unidentified publications that would be of relevance to some specific aspect brought up in this paper.

This paper identifies aspects of ethical relevance in relation to FASD. What it does not do is to settle the relative weights of these different aspects. This lies beyond the scope of this paper. For this purpose more empirical input is needed and also more normative analysis.

The SBU assessment project on FASD, which was the groundwork for this paper, did not evaluate the literature on prevention. In this paper some discussions of prevention are included, but we do not take all aspects of prevention into consideration.

## Conclusions

The aim of this paper was to identify ethical aspects and consequences of diagnostics, interventions, and family support in relation to FASD. FASD covers a broad spectrum of cognitive, physical, behavioral, and social difficulties that affect the everyday lives of concerned individuals and their families – in some cases this impact is considerable and the need for help obvious.

A diagnosis may provide an explanation of the problems and make it easier to get in touch with others experiencing similar difficulties, and to gain access to further evaluation and to resources in the society. However, a diagnosis also comes with a risk for stigmatization, blame, and guilt. Furthermore, it potentially puts an undue stress on the individual for what might be societal problems, such as inequality and social marginalization. It is also problematic from a fairness perspective if individuals with a FASD-related diagnosis obtain access to help from society, while individuals equally badly off but without a diagnosis do not. Hence, there are a number of potential advantages with providing FASD-related diagnoses, but also serious potential drawbacks. Present knowledge is limited regarding the relative weights of these pros and cons.

Furthermore, it is not established whether it is easier for individuals with FASD to receive help and be included in interventions if they obtained a diagnosis, compared to if their separate difficulties are attended to as they are identified. Nor is it established that it is justified to allocate resources based on FASD-related criteria, compared to directing the resources at the most prominent specific needs.

There is also limited knowledge regarding the benefits and harms of specific programs for individuals with FASD conditions and their families. Most existing programs build on training schemes developed for other conditions with similar symptoms – training programs that as such seem ethically unproblematic. More research is needed to determine whether specific interventions could provide more efficient support.

Taken together, it is questionable whether the health and wellbeing of concerned individuals will increase by having their conditions described as FASD-related. However, this picture might change with future development and research on specific supportive interventions.

## Abbreviations

ADHD: Attention Deficit Hyperactivity Disorder
ARBD: Alcohol-Related Birth Defects
ARND: Alcohol-Related Neurodevelopmental Disorder
FAS: Fetal Alcohol Syndrome
FASD: Fetal Alcohol Spectrum Disorders
ND-PAE: Neurobehavioral Disorder associated with Prenatal Alcohol Exposure
PFAS: partial FAS
SBU: Swedish Agency for Health Technology Assessment and Assessment of Social Services
